# Quantification of Veterinary Antibiotics in Pig and Poultry Feces and Liquid Manure as a Non-Invasive Method to Monitor Antibiotic Usage in Livestock by Liquid Chromatography Mass-Spectrometry

**DOI:** 10.3390/molecules25143265

**Published:** 2020-07-17

**Authors:** Ewelina Patyra, Krzysztof Kwiatek, Carolina Nebot, Rosa Elvira Gavilán

**Affiliations:** 1 Department of Hygiene of Animal Feedingstuffs, National Veterinary Research Institute, 24-100 Pulawy, Poland; kwiatekk@piwet.pulawy.pl; 2 Department of Analytical Chemistry, Nutrition and Bromatology, Faculty of Veterinary Medicine, University of Santiago de Compostela, 27002 Lugo, Spain; carolina.nebot@usc.es (C.N.); rosa.elvira@deinal.es (R.E.G.)

**Keywords:** antibacterial substances, residues, feces, non-invasive method, SPE, d-SPE, LC-MS, LC-MS/MS

## Abstract

Antibiotics are active substances frequently used to treat and prevent diseases in animal husbandry, especially in swine and poultry farms. The use of manure as a fertilizer may lead to the dispersion of antibiotic residue into the environment and consequently the development of antibiotic-resistant bacteria. Most pharmaceutical active ingredients are excreted after administration, in some cases up to 90% of the consumed dose can be found in the feces and/or urine as parent compound. Therefore, due to antibiotic metabolism their residues can be easily detected in manure. This article describes a method for simultaneous analysis of ciprofloxacin, chlortetracycline, doxycycline, enrofloxacin, lincomycin, oxytetracycline, tetracycline, tiamulin, trimethoprim and tylosin in feces, liquid manure and digestate. Antibiotics were extracted from the different matrices with McIlvaine-Na_2_EDTA buffer solution and the extract was purified by the use two techniques: d-SPE and SPE (Strata-X-CW cartridges) and final eluent was analyzed by LC-MS and LC-MS/MS. The European Commission Decision 2002/657/EC was followed to conduct the validation of the method. Recoveries obtained from spiked pig and poultry feces and liquid manures samples ranged from 63% to 93% depending on analytes. The analysis of 70 samples (feces, liquid manure and digestate) revealed that 18 samples were positive for the presence of doxycycline, oxytetracycline, tetracycline, chlortetracycline, enrofloxacin, tiamulin and lincomycin. The results obtained in the presented study demonstrated that animal feces can be used as a non-invasive method detection antibiotic usage in animal production.

## 1. Introduction

Antibiotics are substances that can kill or inhibit bacteria growth, they can be produced by microorganisms and synthetically and they are frequently used in both human and veterinary medicine [1]. In animal production, antibiotics are used to treat and prevent animal diseases and in non-European countries antibiotics are used as growth promoters. In Poland and Spain the most commonly applied antibiotics in pig and poultry productions are tetracyclines (chlortetracycline, doxycycline and oxytetracycline), penicillins (amoxicillin), fluoroquinolones (mainly enrofloxacin), sulfonamides (mainly sulfadiazin and sulfamethoxazol) combined with trimethoprim and macrolides (tylosin). Antibiotics are mainly administrated by three different routes: through injection, via feed with medicated feed or via drinking water. After administration of antibiotics between 30% and 90% of the initial dose given is excreted as active metabolites or as non-metabolized form. Therefore, high concentrations of antibiotics and/or their metabolites can be present in urine or feces [2,3]. In general, high percent of the initial dose administrated is excreted unchanged (more than 60%), therefore, in general, antimicrobials are excreted by human and animals as active and/or inactive metabolites [4]. For example, N4-acetylosulfonamides which are the metabolites of sulfonamides are less active compared to the parent drugs. On the other hand, ciprofloxacin is the metabolite of enrofloxacin, which is also antimicrobially active [5,6].

Livestock feces and liquid manure are used as land fertilizer for its high levels of phosphorus, nitrogen and organic matter that can improve the physical and chemical properties of soil and provide essential nutrients to plants [7]. Application of manure as fertilizer is a common practice in many countries including those of the European Union countries. Residues of antibiotics excreted and present in the animal manures/feces enter into the environment either by spreading of livestock wastes onto agricultural fields as fertilizer or in form of sludge after manure collection and storage. Antibiotics present in manures/feces can be a risk for humans and the environment [3].

According to Berendsen and co-workers (2015) analyzing antibiotics in animal feces can help to have knowledge on the dispersion of antibiotics in the environment and their ecotoxicological effects [1]. The study may provide an answer on the emergence of bacterial resistance in the intestines of animals, and, thus, be a valuable source of information on the relationship between antibiotic residues and bacterial resistance. Berendsen and co-workers also indicate that the analysis of animal feces may be useful for monitoring the use of antibiotics on the farm by using a non-invasive sampling and enforce policies on the use of antibiotics and prevent their illegal use [1].

The problem of environmental pollution with antibacterial substances present in organic fertilizers (liquid manure, pig and poultry feces) is becoming more and more recognizable in the world. In recent years, researchers have published several papers demonstrating that antibiotics in feces derived from slaughtered animals are present in high concentrations. In China, organic fertilizers from chickens were analyzed. Researchers found high concentrations of enrofloxacin and norfloxacin of 1420 mg/kg and 225 mg/kg, respectively. In 2007, Carabello and colleagues examined pig droppings in Austria, in which they found the presence of antibiotics from the tetracycline group in amounts of several dozen milligrams per kilogram of feces (tetracycline—23 mg/kg, oxytetracycline—29 mg/kg and chlorotetracycline—46 mg/kg) [8]. The lack of information on the concentration of antibiotic residues in manure applied to agricultural land, with or without processing, is necessary to conduct an adequate environmental risk assessment of veterinary drugs [7].

A few papers have been recently published describing the analysis of different classes of antibiotics in feces or manure. Matrices such as manure and feces are very complex and advanced extraction protocols are required for efficient extraction with high recoveries of the compounds [9]. Commonly used extraction techniques are ultrasonic-assisted [3,8,10,11,12], microwave-assisted [13,14], liquid-liquid [15,16] and accelerated-solvent [17]. Solid-phase extraction is the most popular clean-up technique to avoid interferences with matrix components [18]. High performance liquid chromatography combined with diode array detector, mass spectrometry or tandem mass spectrometry are the methods of choice for the analysis of antibiotics in environmental samples due to its high selectivity and sensitivity [2,3,18,19,20].

The complexity of the manure matrix and the difficulties associated with the analysis of antibiotics demand considerable diligence in sample handling. The present work reports a sensitive and efficient method for the simultaneous extraction and analysis of fluoroquinolones (ciprofloxacin and enrofloxacin), tetracyclines (oxytetracycline, tetracycline, chlortetracycline and doxycycline), lincomycin, tylosin, tiamulin and trimethoprim in chicken and pig feces, liquid manure and digestate using dispersive solid phase extraction and solid-phase extraction and liquid chromatography–mass spectrometry. The validated method was applied to analyzing real liquid manure, feces and digestate samples collected from biogas factories and pig and poultry farms located in Poland and Spain.

## 2. Results and Discussion

### 2.1. LC Analysis

Samples were analyzed using LC-MS and LC-MS/MS. MS and MS/MS parameters were optimized by infusing individual solutions of the analytes at 1 µg/mL into each instrument. All analytes showed acceptable sensitivity in the positive mode (ESI+). According to Commission Decision 2002/657/EC [21] for multiple reaction monitoring (MRM) two transitions are required for each compound to be able to reach the minimum number of identification points to avoid unambiguous confirmation. In the presented work two MRM transitions for reliable confirmation were possible for all the compounds analyzed. Chromatographic conditions were optimized to improve separation, sensitivity and selectivity taking into account the compound investigated.

**Figure 1:**
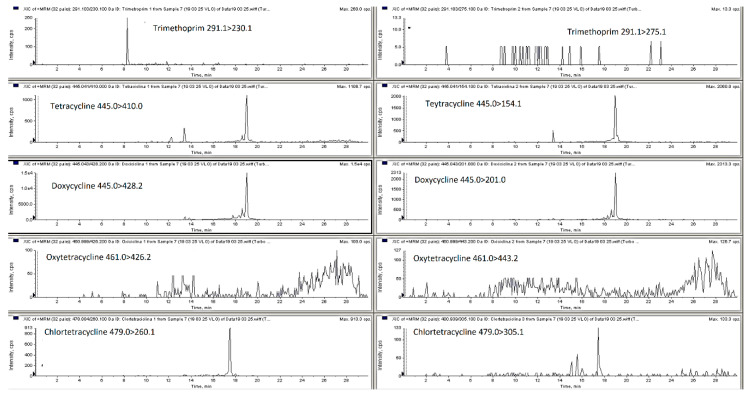

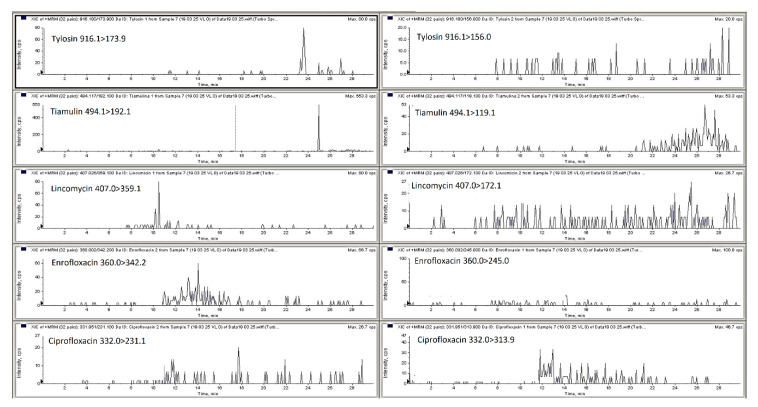
LC-MS/MS MRM chromatograms of blank pig feces.

**Figure 2:**
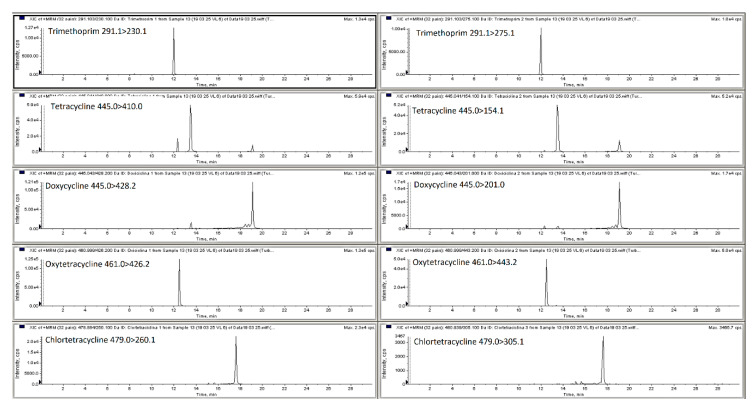

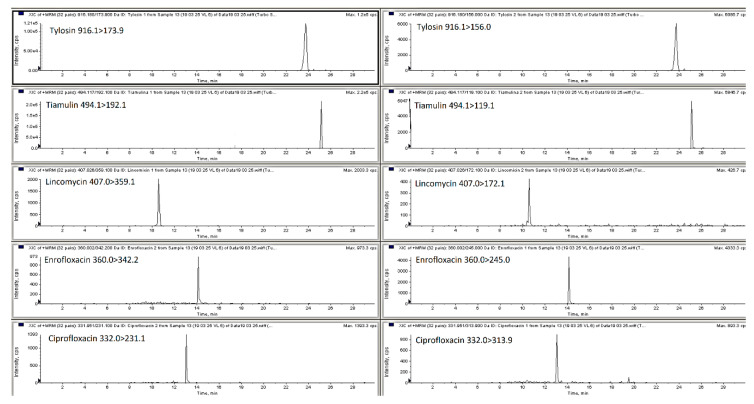
LC-MS/MS MRM chromatograms of spiked pig feces sample with all analyzed compounds at first validation level (trimethoprim—25 µg/kg; oxytetracycline, tetracycline, chlortetracycline, doxycycline, tylosin, tiamulin—100 µg/kg; lincomycin—125 µg/kg; enrofloxacin, ciprofloxacin—50 µg/kg).

The mobile phase optimization was necessary to obtain satisfactory response for the different compounds at the different concentration levels and for each type of matrix selected (poultry and pig feces, liquid manure and digestate). For the analysis of antibacterial substances from liquid manure, animal feces and soil matrices researchers have employed a mobile phase ammonium acetate, formic acid, ammonium formate in water combination with methanol or acetonitrile with or without formic acid, ammonium formate or ammonium acetate [1,2,18,22,23,24,25]. Thus, in this research elution was achieved with a gradient with 0.1% of formic acid in water and 0.1% of formic acid in acetonitrile, this mixture permitted obtaining satisfactory retention times and good peaks shape. The chromatography separation of the different classes of antibacterial from liquid manure or animal feces conducted by other researchers is usually performed employing C_18_ chromatographic columns such as Nucleosil C_18_ HD, Kinetex C_18_, Genesis C_18_ and ACQUITY UPLC BEH C_18_ [1,2,8,19,22,25,26]. In this particular work, three HPLC columns were compared a Phenomenex Kinetex C18 (100 × 2.6 mm, 5 μm), Agilent Zorbax Eclipse C18 (150 × 4.6 mm, 5 µm) and Phenomenex Luna C18 (150 × 4.6 mm, 5 µm). The Kinetex C18 column and mobile phase consisting of 0.1% formic acid in Milli-Q water and 0.1% formic acid in acetonitrile gave the best results (peak resolution and signal intensity) with an injection volume of 15 µL. Figure 1 and Figure 2 show MRM chromatograms of blank pig feces sample and pig feces sample spiked with all analyzed compounds at the first validation level (see Section 3.7). All samples were analyzed using liquid chromatography with a single and tandem mass spectrometer.

**Figure 3:**
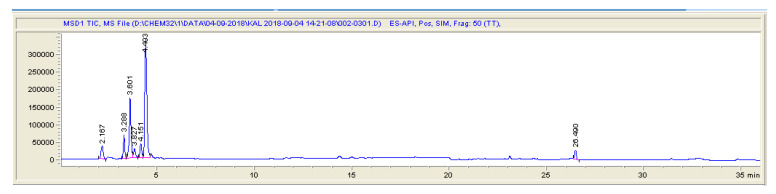
LC-MS (SIM) chromatogram of blank pig feces sample.

**Figure 4:**
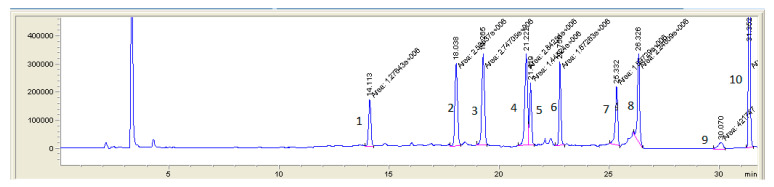
LC-MS (SIM) chromatogram of pig feces sample spiked with all analyzing antibacterial substances at first validation level (1—lincomycin 125 µg/kg; 2—trimethoprim 25 µg/kg; 3—oxytetracycline 100 µg/kg; 4—ciprofloxacin 50 µg/kg; 5—tetracycline 100 µg/kg; 6—enrofloxacin 50 µg/kg; 7—chlortetracycline 100 µg/kg; 8—doxycycline 100 µg/kg; 9—tylosin 100 µg/kg; 10—tiamulin 100 µg/kg).

The obtained results showed that both LC-MS and LC-MS/MS systems are useful for analysis of animal feces, liquid manure and digestate samples in the same concentration ranges, using the same chromatographic column and sample preparation method. A typical chromatograms for both methods are shown in Figure 1, Figure 2, Figure 3 and Figure 4.

### 2.2. Extraction Procedure

The extraction of antibiotics from liquid manure and animal feces can be difficult for the presence of high amounts of natural organic matter present in animal feces. Extraction of antibacterial substances from this kind of matrices was optimized through a set of different experiment aimed to maximize recoveries and achieve the lowest limit of quantification. It was taken into account that protocols of extraction reported previously for antibiotics analysis from different therapeutic groups detected in liquid manure and animal feces employed different extraction procedures. The following factors were investigated: sample weight (10 g, 5 g and 2 g), extraction solvent (methanol and McIlvaine-Na_2_EDTA and McIlvaine-Na_2_EDTA buffer solution at pH 4 and 7) and extraction techniques. To extract the antibiotic from the matrix two techniques were compared: shaking using an orbital shaker and ultrasonification. Because manure and feces are very complexity matrices, ultrasonic extraction was used first to improve the extraction of antibiotics bound to the natural organic matter, waves cause cavitation and high shear forces within the matrix what allow mass transfer to the extraction solvent [27]. The use of these extraction mixtures and extraction methods is an insufficient process for analyzing antibiotics in liquid manure, feces and digestate. The obtained extracts require further purification process. For this purpose, we used tandem-SPE technique, described by Blackwell et al. (2004), first [19]. For slurry extraction procedure authors used mixture 0.1 M EDTA and McIlvaine buffer at pH 7. After extraction, slurry samples were acidified with 50 µL of H_3_PO_4_. The extract was transferred to SAX-HLB SPE cartridges connected in tandem and pre-conditioned with methanol and buffer. The diluted extract was passed through the cartridges. The SAX cartridges were removed, HLB cartridges were washed sequentially with 0.1 M EDTA-McIlvaine buffer pH 2.9, sodium acetate, distillate water and 20% methanol. Antibiotics were eluted from the HLB cartridges with 4 mL of methanol. The extracts were analyzed and unsatisfactory results were obtained as detection of ciprofloxacin, enrofloxacin, tiamulin and lincomycin was not achieved. In addition, the detection limit for tetracyclines was approximately 300 µg/kg, for tylosin and trimethoprim 250 µg/kg in feces and 200 and 100 µg/kg in liquid manure and digestate. Therefore, the method was improved, in the second stage of this study we used the extraction protocol described by Patyra and Kwiatek (2017) [28]. Authors extracted tetracyclines from feed using McIlvaine-Na_2_EDTA buffer pH 4 using orbital shaker and clean up step with the use Strata-X-CW cartridges. The extracts were reduced with a stream of nitrogen and diluted in 0.1% formic acid in Milli-Q water and analyzed by LC-MS. The supernatant was colored due to the presence of natural organic materials. It is important to reduce organic materials, as high amounts could be accumulated inside the liquid chromatography column and electrospray source leading to signal reduction after only a few injections. Therefore, again, the extraction protocol was not satisfactory as a large matrix effect and impurities from analyzing matrices were observed, the applied method of extraction and purification allowed the detection of all the analyzed antibiotics: Oxytetracycline, tetracycline, chlortetracyline, doxycycline, ciprofloxacin, enrofloxacin, trimethoprim, tylosin, tiamulin and lincomycin.

To remove colorants from the sample extract and pre-purification, the first dispersive solid phase extraction was used. For this purpose, a mixture of sorbent consisting of activated carbon, C_18_ and PSA sorbents. Then the pre-purified d-SPE technique extracts were then purified by SPE using Strata-X-CW cartridges.

The experiments shown that the purest extracts and the best recoveries were obtained employing a combination of McIlvaine-Na_2_EDTA buffer at pH 4 in combination with a clean-up protocol using d-SPE and SPE Strata-X-CW cartridges. To the best of our knowledge, this is the first method described, which uses a combination of two purification techniques to analyze antibacterial substances in liquid manure, animal feces and digestate.

### 2.3. Method Validation

The whole procedure was validated according to the requirements of the European Union regulation 2002/657/EC [21].

Linearity was evaluated by the use of matrix matched calibration curves for each compound. Matrix matched calibration curves were constructed using analyte peak area versus concentration analyte. The coefficient of determination (R^2^) for the six-point calibration curves above 0.98. The specificity of the method was tested by processing and analyzing 20 different control feces and liquid manure samples.

The accuracy and precision of the method in terms of the intra- and inter-day precision and recovery of the assay, feces and liquid manure samples at the three concentration levels were prepared. The intra-day precision was assessed by calculating CV for six replicates and inter-day precision was determined with the results of the s of samples on three consecutive days. The mean recovery ranged between 63.2% and 93.5% for all analytes. Recovery was obtained for the target analytes employed results at all spiked levels (Table 1). The intra- and inter-day precision for the target analytes were lower than 21% and 29%, respectively, at all spiking levels.

The values of CCα and CCβ of the method were determined. The CCα and CCβ values ranged from 23.2 to 101.8 µg/kg and 38.0–168.2 µg/kg, respectively. The LOD was taken as a S/N of 3:1 and the LOQ was defined as a S/N of 10:1. All parameters are presented in Table 1. The method was shown to be appropriate for all the analytes with acceptable accuracy and precision.

### 2.4. Application to Feces, Liquid Manure and Digestate Samples 

The samples were collected from two country: Poland and Spain. A total of 70 samples were collected, including 49 pig feces, 3 poultry manure, 4 pig liquid manure and 14 digestate from biogas factory. Each sample was determined by the method established in this work. The most often antibacterial substance detected in the analyzed samples was doxycycline. Doxycycline was found in 14 feces and 1 liquid manure samples and the concentrations were in the range 200–175,400 µg/kg. Oxytetracycline, tetracycline and chlortetracycline were detected in 4, 2 and 2 samples, respectively, and concentrations were between 220 and 1450 µg/kg, 1320–1710 µg/kg and 340–17,700 µg/kg, respectively. Pig feces and digestate samples contained residues of taimulin, lincomycin and enrofloxacin. Furthermore, mixtures of antibiotics were found (three and two antibiotics in feces taken from a single animals). Positive feces, liquid manure and digestates samples are shown in Table 2.

## 3. Materials and Method

### 3.1. Chemicals and Reagents 

Oxytetracycline (OXT), chlortetracycline (CTC), tetracycline (TC), doxycycline hyclate (DC), tiamulin fumarate (TIAM), tylosine tartrate (TYL), ciprofloxacin (CIP), enrofloxacin (ENR), lincomycin (LINCO), trimethoprim (TRIM), activated carbon, EDTA disodium salt and formic acid were obtained from Sigma Aldrich (St. Louis, MO, USA). HPLC-grade methanol, acetonitrile and octadecyl C_18_ 40 µm Prep LC sorbent were purchased from Baker (Deventer, The Netherlands). Disodium hydrogen phosphate and sodium hydroxide were from Chempur (Piekary Śląskie, Poland), citric acid was from Acros Organic (Morris Plains, NJ, USA) and PSA Bonded Silica sorbent was from Supelco (Bellefonte, PA, USA). Purified water was prepared with a Milli-Q water system from Millipore (Billerica, MA, USA).

McIlvaine-Na_2_EDTA buffer was prepared by dissolving 11.406 g Na_2_EDTA in 115.65 mL 0.2 M phosphate buffer and 184.65 mL 0.1 M citric acid. The pH was adjusted to 4.0 by adding 0.1 M citric acid or 0.2 M phosphate buffer. Three SPE cartridges were tested; OASIS HLB (60 mg, 3 mL) from Waters (Milford, MA, USA) and Strata-SAX (strong anion exchanger, 500 mg, 12 mL) and Strata-X-CW (weak cation mixed mode, 300 mg, 3 mL) both from Phenomenex (Torrance, CA, USA). An SPE manifold (J.T. Baker, PA, USA) and a pump as a vacuum source were used.

### 3.2. Preparation of Standard Solutions

A portion of 10 mg of reference antibiotic was dissolved into 10 mL of solvent, concentration was calculated taking in consideration the purity of the compound. Lincomycin, tylosin, tiamulin, trimethoprim, enrofloxacin, oxytetracyline, tetracycline, chlortetracycline and doxycycline were dissolved in methanol, and ciprofloxacin in methanol:1 M NaOH (99:1; *v/v*). A standard working solution of each antibiotic was prepared freshly each day by diluting stock solutions to final concentrations of 100 μg/mL and 10 μg/mL.

### 3.3. LC-MS

The LC-MS system was composed by an Agilent 1200 series liquid chromatograph which consisted on a binary pump, a degasser, an autosampler, a column heater coupled to a single quadrupole mass spectrometer from Agilent 6140 (Agilent Technology, Santa Clara, CA, USA). Positive electrospray mode employed for all analytes and detection was performed with selected ion monitoring. The ChemStation software also from Agilent Technology controlled the LC-MS system and processed the data. The operating parameters were drying gas temperature (350 °C), drying gas flow (12 L/min), nebulizing gas pressure (35 psi) and capillary voltage 2000 V. Molecular masses of the precursor ions of all detection antibiotics was shown in Section 3.4. The separation of the antibacterial substances was performed on a Kintex octadecyl C_18_ (100 × 2.6 mm, 5 μm) column protected by a RP_18_ guard column (4.0 × 3.0 mm, 5 μm), both from Phenomenex, operated at 25 °C. The mobile phase consisted of 0.1% formic acid in Milli-Q water (solvent A) and 0.1% formic acid in acetonitrile (solvent B). The gradient used was 0–1 min, 5% B; 1–15 min, 15% B; 15–26 min, 36% B; 26–29 min, 100% B; 29–30 min, 100% B; before returning to 5% B in 1 min, with a final hold at 5% B until 36 min. The flow rate was 0.4 mL/min and the injection volume was 15 μL.

### 3.4. LC-MS/MS

The 1100 HPLC separation module with pump, degasser, column oven and autosampler all from Agilent Technologies (Waldbronn, Germany) coupled to a Qtrap 2000^TM^ mass spectrometer from Applied Biosystems/MDX-Sciex (Toronto, ON, Canada) was the LC-MS/MS system employed which was controlled by the Analyst 1.4.1 software also from Apllied Biosytems. The chromatographic analyses ware performed by injecting 15 µL of extract into a Kinetex C18 column. Mobile phase A and B consisted of 0.1% formic acid in water (A) and 0.1% formic acid in acetonitrile (B). Gradient program is presented in Table 3.

Mass spectrometry (MS) measurement were performed using electrospray source in positive mode (ESI+). Selected veterinary drugs were identified by their retention times (*t*_R_) and multiple reaction monitoring. The MS conditions employed to monitor each transition are summarized in Table 4. Stock solutions of individual drugs at 1 µg/mL were analyzed to verify MRM transitions and *t*_R_ selected.

Specific MS parameters optimized during the method development where needed for each MRM transitions; entrance potential (EP), collision cell entrance potential (CEP), cell exit potential (CXP), collision energy (CE) and declustering potential (DP). These parameters differ from one compound to another and change during the run. The dwell-time employed between transitions was 150 ms.

General MS parameters during antibiotic analysis were: A source temperature of 450 °C, an ion spray voltage of 5500 V, a vacuum gauge of 2.2 atm and a curtain gas of 1.2 × 10^4^ Pa, ion source 1 was set at 2.6 × 10^4^ Pa and ion source 2 at 2.4 × 10^4^ Pa. These parameters were set during the whole run.

### 3.5. Sample Preparation and Extraction

An aliquot of sample (liquid manure, feces or digestate) of 5 ± 0.01 g was accurately weighted and introduced into a 50 mL centrifuge tubes. Antibiotics were added to control samples and shaken for 30 s on a vortex mixer, they were kept in the dark for 3 h for equilibration. Then 25 mL of McIlvaine-Na_2_EDTA buffer was used to extract pharmaceutical residues. At room temperature the samples shaken for 45 min on a horizontal shaker and centrifuged at 4000 rpm for 20 min. 

### 3.6. Clean-up

For the clean-up step, first dispersive solid phase extraction (d-SPE) was employed. To the 50 mL Falcon tubes 300 mg of PSA, 300 mg of C_18_ sorbents and 50 mg of activated carbon was added. Them, the supernatant obtained in the extraction step was transferred to the Falcon tube containing d-SPE. The mixture was shaken at room temperature for 15 min on a horizontal shaker and centrifuged at 4000 rpm for 20 min at 20 °C. After centrifugation extract was filtered through a paper filter (Whatman, No 1, Camlab, UK).

In the second clean-up step, extract was loaded on a Strata-X-CW cartridge (300 mg, 3 mL) which was activated with 3 mL of methanol, and 3 mL of water. After percolation, the cartridge was washed with 6 mL of water, 6 mL of methanol and 3 mL of acetonitrile and vacuum dried for 5 min. The analyte was eluted with 3 mL of a mixture of 2% of formic acid in methanol. The eluate was evaporated to dryness under a stream of nitrogen at 40 °C ± 5 °C. The dry extracts were resuspended in 600 µL 0.1% formic acid in Milli-Q water, vortex mixed, and transferred into vials for analysis.

### 3.7. Method Validation 

The Commission Decision 2002/657/EC and ICH guideline were followed to validate the method. Method linearity, limits of detection (LOD) and limit of quantification (LOQ), decision limit (CCα), detection capability (CCβ), method repeatability, within-laboratory reproducibility and recovery were evaluated by spiking experiments using sample material that was tested free from analyzing antibacterial substances. The specificity of the method was assessed through the analysis of unspiked blank samples.

Linearity was evaluated by constructing the calibration curves by analyzing liquid manure and feces spiked with the standard solutions. The calibration curves were constructed by plotting the peak area of the analyte versus nominal concentration (concentration levels for all analyzing substances are shown in Table 5). LOD and LOQ values were the concentrations of analytes in a matrix-matched samples which gave a signal-to-noise (S/N) higher than three and ten, respectively. In the selectivity/specificity study, 20 blank pig and poultry feces, liquid manure and digestate were analyzed. For inter-day and intra-day precision a set of six replicates at three concentration levels (Table 6) was used. For each concentration level standard deviations (SD) and coefficients of variation (CV, %) were calculated. The recoveries were evaluated by comparing the measured concentrations with the fortified concentrations of the samples.

Decision limits and detection capability were calculated using within-laboratory reproducibility results, according to the following Equations (1) and (2):

CCα = 1st spiking level + 2.33 × SD_WRL_ (1)

CCβ = CCα + 1.64 × SD_WRL_ (2)

where SD_WRL_ is a standard deviation of within-laboratory reproducibility at first spiking level.

### 3.8. Sample Collection 

In 2018, 23 pigs’ feces, 4 pigs liquid manure, 3 chicken manure and 14 digestate samples were collected from different regions of Poland. In March 2019, 26 pig feces samples were collected from Galicia pig farms, Spain. Poultry manure consists of the mixture of urine, feces litter generated on poultry farms. Liquid manure is a liquid, fermented mixture of feces (feces and urine) of farm animals and water, possibly with an admixture of unused feed, which is collected in tanks. Pig liquid manure samples were taken directly from tanker lorries and pig feces samples were directly from the pigsty. Digestate came from biogas factory in Poland. Digestate is a residue from the anaerobic digestion of plant and animal manure. It consists of biomass of methane bacteria, unfermented organic compounds and minerals. Digestate can be used as organic fertilizer on farmland. All samples were stored in plastic containers and refrigerated at −20 °C until analysis.

## 4. Conclusions

A sensitive method for determination of oxytetracycline, tetarcycline, chlortetracycline, doxycycline, ciprofloxacin, enrofloxacin, lincomycin, tiamulin, trimethoprim and tylosin in pig feces, poultry manure, liquid manure and digestate from biogas factory has been developed. Sample preparation was performed using two clean-up steps: Dispersive solid phase extraction and solid phase extraction with Strata-X-CW cartridges followed by analyzing HPLC-MS/MS. The analysis of 70 samples revealed that 18 samples (25.7%) were positive for the presence of antibacterial substances. Furthermore, mixtures of antibiotics were found (three and two antibiotics in feces taken from a single animals). Analysis of manure is a simple and non-invasive strategy to monitor antibiotic use in animal breeding and useful to understand antibiotic dispersion and possible development resistant bacteria in the environment. The results presented demonstrated that measured veterinary antibacterial substances can lead to the contamination of agricultural soils via fertilization through manure.

## Figures and Tables

**Table 1:** Validation parameters of the optimized method.

Analyte	Linearity	Recovery [%]			Reproducibility [%]			Reproducibility [%]			LOQ [µg/kg]	LOD [µg/kg]	CCα [µg/kg]	CCβ [µg/kg]
		Concentration Levels [µg/kg]			Concentration Level [µg/kg]			Concentration Level [µg/kg]						
		I	II	III	I	II	III	I	II	III				
CIP	50–1000 µg/kg	64.4	66.1	60.3	21.0	19.8	20.0	26.9	28.8	27.1	34.9	21.2	46.0	98.2
ENR	50–1000 µg/kg	75.1	81.0	83.9	15.5	16.2	15.2	18.1	19.0	21.0	30.8	19.5	35.2	53.3
OXT	100–1500 µg/kg	89.1	92.2	93.5	10.0	10.7	8.8	18.4	13.7	12.4	61.0	54.3	77.9	111.7
TC	100–1500 µg/kg	82.0	81.0	86.7	16.1	17.7	13.6	19.4	20.5	15.4	73.0	60.7	86.4	161.0
CTC	100–1500 µg/kg	84.5	80.9	82.2	11.4	11.0	8.9	15.4	13.9	18.9	78.8	64.3	76.8	120.2
DC	100–1500 µg/kg	74.3	79.1	75.8	17.9	12.3	14.1	21.9	16.0	15.3	87.2	69.1	89.0	156.2
TYL	100–1500 µg/kg	63.2	65.3	64.3	22.2	14.0	12.1	28.7	17.7	19.9	82.3	67.9	91.1	168.2
TIAM	100–1500 µg/kg	88.4	88.0	91.4	11.3	10.7	12.2	13.5	15.3	12.1	45.5	39.3	53.2	78.8
LINCO	125–625 µg/kg	75.5	72.3	81.0	11.0	10.2	9.9	14.5	13.1	13.0	92.0	76.2	101.8	133.5
TRIM	25–500 µg/kg	90.0	100.2	93.3	9.8	7.8	5.6	6.8	7.0	8.4	15.5	10.2	23.2	38.0

**Table 2:** The analytes and their concentrations detected in real samples.

Sample	Analyte [µg/kg]									
	CIP	ENR	OXC	TC	CTC	DC	TYL	TIAM	LINCO	TRIM
PIG FAECES										
Samples from Poland										
S1	nd	nd	nd	nd	nd	200	nd	nd	nd	Nd
S2	nd	nd	nd	nd	nd	200	nd	nd	nd	Nd
S3	nd	nd	1450	nd	nd	4141	nd	nd	nd	Nd
S4	nd	nd	nd	nd	nd	980	nd	nd	nd	Nd
S5	nd	nd	nd	nd	nd	1900	nd	nd	nd	Nd
S6	nd	nd	nd	nd	nd	1165	nd	nd	nd	Nd
Samples from Spain										
S7	nd	nd	nd	nd	340	1000	nd	nd	nd	Nd
S8	nd	nd	nd	nd	nd	34,340	nd	nd	nd	Nd
S9 *	nd	nd	440	nd	nd	175,400	nd	520	nd	Nd
S10	nd	nd	220	nd	nd	125,140	nd	nd	290	Nd
S11	nd	nd	nd	nd	nd	5540	nd	nd	nd	Nd
S12	nd	nd	nd	1710	17,700	nd	nd	nd	nd	Nd
S13 *	nd	nd	410	nd	nd	97,900	nd	nd	nd	Nd
S14	nd	nd	nd	1320	nd	16,540	nd	nd	nd	Nd
S15	nd	nd	nd	nd	nd	18,340	nd	nd	nd	Nd
LIQUID MANURE										
M1	nd	nd	nd	nd	nd	5900	nd	nd	nd	Nd
DIGESTATE										
D1	nd	nd	nd	nd	nd	nd	nd	148	nd	Nd
D2	nd	50	nd	nd	nd	nd	nd	nd	nd	Nd

* Feces samples from animals during treatment; S9—animals treated with doxycycline and tiamulin in water; S13—animals treated with doxycycline.

**Table 3:** Gradient program.

Step	Total Time (min)	Flow Rate (µL/min)	A (%)	B (%)
0	0	400	100	0
1	1	400	100	0
2	5	400	85	15
3	15	400	74	26
4	20	400	64	36
5	24	400	0	100
6	25	400	100	0
7	31	400	100	0

**Table 4:** Precursor and product ions, declustering potential (DP), collision energy (CE), entrance potential (EP), cell exit potential (CXP) and collision cell entrance potential (CEP) optimal conditions employed for MS identification of each compound.

Analyte	Precursor Ion	Production	DP	EP	CEP	CE	CXP
Lincomycin 1	407.0	359.1	31	7.5	18.29	17	6
Lincomycin 2	407.0	172.1	31	7.5	18.29	25	4
Trimethoprim 1	291.1	230.1	31	12	14.15	33	8
Trimethoprim 2	291.1	275.1	31	12	14.15	33	12
Oxytetracycline 1	461.0	426.2	26	10	20.22	21	6
Oxytetracycline2	461.0	443.2	26	10	20.22	21	18
Doxycycline 1	445.0	428.2	31	10	19.65	21	6
Doxycycline 2	445.0	201.0	31	10	19.65	57	4
Tetracycline 1	445.0	410.0	41	8	19.65	25	15
Tetracycline 2	445.0	154.1	41	8	19.65	35	5
Enrofloxacin 2	360.0	342.2	56	10	16.61	33	6
Enrofloxacin 1	360.0	245.0	56	10	16.61	39	6
Chlortetracycline 1	479.0	260.1	51	9.5	20.86	73	4
Chlortetracycline 2	479.0	305.1	51	10	20.94	39	6
Ciprofloxacin 2	332.0	231.1	91	8.5	15.61	41	8
Ciprofloxacin 1	332.0	313.9	91	8.5	15.61	35	6
Tylosin 1	916.2	173.9	81	9	36.5	49	4
Tylosin 2	916.2	156.0	81	9	36.5	51	4
Tiamulin 1	494.1	192.1	41	8	21.41	23	4
Tiamulin 2	494.1	119.1	41	8	21.41	23	4

**Table 5:** Concentration levels of matrix match calibration curves for all analyzed compounds.

	Concentration Levels [µg/kg]									
	CIP	ENR	OXT	TC	CTC	DC	TYL	TIAM	LINCO	TRIM
1	0	0	0	0	0	0	0	0	0	0
2	50	50	100	100	100	100	100	100	125	25
3	100	100	200	200	200	200	200	200	250	50
4	200	200	500	500	500	500	500	500	375	100
5	500	500	1000	1000	1000	1000	1000	1000	500	250
6	1000	1000	1500	1500	1500	1500	1500	1500	625	500

**Table 6:** Validation spiking levels.

	Spiking Levels [µg/kg]									
	CIP	ENR	OXT	TC	CTC	DC	TYL	TIAM	LINCO	TRIM
**I**	50	50	100	100	100	100	100	100	125	25
**II**	200	200	500	500	500	500	500	500	375	100
**III**	1000	1000	1500	1500	1500	1500	1500	1500	625	500
